# *FUT11* as a potential biomarker of clear cell renal cell carcinoma progression based on meta-analysis of gene expression data

**DOI:** 10.1007/s13277-013-1344-4

**Published:** 2013-12-08

**Authors:** Elżbieta Zodro, Marcin Jaroszewski, Agnieszka Ida, Tomasz Wrzesiński, Zbigniew Kwias, Hans Bluyssen, Joanna Wesoly

**Affiliations:** 1 Laboratory of High Throughput Technologies, Institute of Biotechnology and Molecular Biology, Faculty of Biology, Adam Mickiewicz University, Umultowska 89, 61-614 Poznań, Poland; 2 Department of Human Molecular Genetics, Institute of Biotechnology and Molecular Biology, Faculty of Biology, Adam Mickiewicz University, Umultowska 89, 61-614 Poznań, Poland; 3 Department of Urology and Urologic Oncology, Poznan University of Medical Sciences, Fredry 10, 61-701 Poznań, Poland

**Keywords:** Biomarker, Gene expression, Meta-analysis, Renal cell carcinoma

## Abstract

In this paper, we provide a comprehensive summary of available clear cell renal cell carcinoma (ccRCC) microarray data in the form of meta-analysis of genes differentially regulated in tumors as compared to healthy tissue, using effect size to measure the strength of a relationship between the disease and gene expression. We identified 725 differentially regulated genes, with a number of interesting targets, such as *TMEM213*, *SMIM5*, or ATPases: *ATP6V0A4* and *ATP6V1G3*, of which limited or no information is available in terms of their function in ccRCC pathology. Downregulated genes tended to represent pathways related to tissue remodeling, blood clotting, vasodilation, and energy metabolism, while upregulated genes were classified into pathways generally deregulated in cancers: immune system response, inflammatory response, angiogenesis, and apoptosis. One hundred fifteen deregulated genes were included in network analysis, with *EGLN3*, *AP-2*, *NR3C1*, *HIF1A*, and *EPAS1* (gene encoding HIF2-α) as points of functional convergence, but, interestingly, 610 genes failed to join previously identified molecular networks. Furthermore, we validated the expression of 14 top deregulated genes in independent sample set of 32 ccRCC tumors by qPCR and tested if it could serve as a marker of disease progression. We found a correlation of high fucosyltransferase 11 (*FUT11*) expression with non-symptomatic course of the disease, which suggests that *FUT11*'s expression might be potentially used as a biomarker of disease progression.

**Electronic supplementary material:**

The online version of this article (doi:10.1007/s13277-013-1344-4) contains supplementary material, which is available to authorized users.

## Introduction

Renal cell carcinoma (RCC) is the most common type of kidney cancer that accounts for 2 % of the world total of all adult malignancies. Its most frequent histological subtype—clear cell renal cell carcinoma (ccRCC)—constitutes 75 % of all kidney tumors with 209,000 new cases per year worldwide [1]. ccRCC arises from the renal cortex, and its lipid- and glycogen-rich cells are “clear” on hematoxylin and eosin staining. ccRCC may have sporadic (>96 %) or familial (<4 %) origin (VHL syndrome) [2]. The majority of ccRCC cases are detected incidentally by ultrasound, CT scan, or MRI, and are diagnosed at the late stage due to asymptomatic course of the disease. The classic symptoms such as hematuria, flank pain, fatigue, and abdominal mass occur rarely and are generally indicative of a more advanced disease [3]. ccRCC is difficult to treat and rarely cured once spread beyond the kidney [4]. If limited to kidneys (40 % of diagnosed cases), the most common curative treatment remains a radical or partial nephrectomy [5]. In advanced stages, targeted (immuno- and antiangiogenic) therapy is introduced. Kinase and mammalian target of rapamycin inhibitors were used in a number of clinical trials and evaluated in the aspect of prognosis improvement [6]. However, their long-term clinical effect remains to be determined and requires larger, homogenous, and well-designed retrospective studies.

In the last decade, a large number of markers has been studied for their prognostic value in ccRCC such as carbonic anhydrase IX, p53, XIAP, HIF1-α, VEGF, and Survivin, but their clinical use remains debatable [7]. The vast majority of research is focused on mechanisms which are deregulated and well described in various cancers such as cell differentiation, angiogenesis, and immunosuppression, but there is a need for identification of targets or pathways unique to ccRCC. A better understanding of molecular pathogenesis of ccRCC is required to direct novel therapeutic intervention of the individual patient and to predict patient's prognosis. Several genomic alterations were suggested to be associated with ccRCC tumorgenesis; however, currently there are no accepted molecular biomarkers to monitor ccRCC development [8].

Molecular markers could be incorporated into future staging systems and hold great promise for more accurate prognoses of ccRCC. Advances in technology, such as gene arrays and high-throughput tissue arrays, make the detection of such markers more visible [3].

However, single microarray studies suffer from several problems: they may report findings not reproducible or not robust to data perturbations, [9–11]. Several meta-analysis techniques have been proposed in the context of microarrays so far; however, a comprehensive framework on how to carry out a meta-analysis of microarray data set emerged only recently [12].

In this paper, we provide the results of a meta-analysis of nine selected ccRCC studies using effect size as a measure of the strength of a relationship between two variables (here: the disease and expression of a gene). The goal of this study was to identify genes that are differentially expressed between ccRCC and normal tissue, to group them according to their function (pathway analysis) and to validate a number of potential biomarkers in a homogenous patient group, well defined with respect to *VHL*, *HIF1A*, *EPAS1* expression and clinical parameters. In order to test prognostic value of the most deregulated genes, we performed logistic regression analyses of clinical and molecular parameters, and showed an association of high expression of the fucosyltransferase gene (*FUT11*) with non-symptomatic disease course up to 31 months post-surgery.

## Material and methods

### Study selection and data set preparation

Twelve Affymetrix studies of biopsy confirmed, primary ccRCC samples with TNM, F grades, or WHO classifications were included. The data, in the FLEO format, were obtained from ArrayExpress (http://www.ebi.ac.uk/arrayexpress/) [13] and Gene Expression Omnibus (http://www.ncbi.nlm.nih.gov/geo/) [14]. There were no technical replicates, no information regarding batch effects or image files. For each study, array density plots, MA plots, Spearman correlation plots, and RNA degradation plots were created to reject low quality arrays. Arrays were normalized using the Robust Multichip Average method [15]. Eight studies fulfilled inclusion criteria and 222 tumor and 85 control samples were subjected to the analysis. For each array type, the probes were mapped to version 14 Unigene gene identifiers (Microarray Lab of the University of Michigan, http://brainarray.mbni.med.umich.edu/brainarray/Database/CustomCDF/) [16].

### Estimation of a study-specific differential expression of each gene

Effect size was used to measure differential expression of each gene using the following formula:

$$ {\varTheta}_g=\frac{\mu_1-{\mu}_2}{\sigma_g}J $$

with *μ*_1_ is the average signal intensity in tumor samples, *μ*_2_ the average signal intensity in controls, *σ*_*g*_ pooled standard deviation, and *J* a constant. Variance *ω*_*g*_ of *θ*_*g*_ was used as a weight while combining study-specific estimates of differential expression of each gene into a single effect size value.

### Combination of study-specific estimates into a single statistic

All genes that were found in less than four studies were removed. An inverse variance technique was used to combine study-specific effect size values into a weighted average, and, for each gene, the following formula was used (*k* is the number of studies):

$$ {\varTheta}_g=\frac{\frac{\theta_{g1}}{\omega_{g1}}+\frac{\theta_{g2}}{\omega_{g2}}+\dots +\frac{\theta_{gk}}{\omega_{gk}}}{\omega_{g1}\kern0.5em +{\omega}_{g2}+\dots +{\omega}_{gk}} $$

*p* value of the summary effect size was calculated and adjusted for multiple testing using the FDR method.

### Data analysis

Computations were performed using the R software (www.r-project.org) and the BioConductor package (http://www.bioconductor.org/). Output genes were converted to Ensemble and Entrez gene ID formats with SOURCE (http://source.stanford.edu) and Biomart (http://central.biomart.org/) ID converters. With the aid of SOURCE or GeneCards Human Gene Database (http://genecards.org), they were also annotated with location, function, and Gene Ontology terms (http://www.geneontology.org/) (Gene Ontology Consortium). The genes were subject to the Gene Functional Classification tool of the Database for Annotation, Visualization and Integrated Discovery (http://david.abcc.ncifcrf.gov/home.jsp) [17]. Correlation and logistic regression analyses were performed using IBM SPSS Statistics 21.

### Patient material

Tumors were collected from patients from Western Poland who were diagnosed with urological carcinomas. In one case, two tumors were detected (patient 01–068) and both tumors were tested for expression of selected genes. The tissues were histopatologically verified as ccRCC and screened for *VHL* mutations, promoter methylation, expression of *VHL*, *HIF1A* and *EPAS1*, and LOH (data not shown). The study was approved by the local ethical committee (876/09); only patients who signed written consent were included in the study. Disease progression was defined as local disease recurrence or distant metastasis detected by X-ray and abdominal ultrasound, and/or abdominal and pectoral CT. Follow-up time of the patients differs per case. In general, first follow-up visits were carried out approximately 6 or 12 months post-nephrectomy. For detailed patient characteristics, see Online Resource 1, Table S1. The control samples comprised of nine histopathologically unchanged tissues matched to 9 of 32 tumors tested.

### qPCR

Primers were designed using Primer-BLAST (www.ncbi.nlm.nih.gov/tools/primer-blast) and Oligo Analyzer 3.1 (http://eu.idtdna.com/analyzer/applications/oligoanalyzer/default.aspx). One microgram of RNA was reversely transcribed using RevertAid™ First Strand cDNA Synthesis Kit with Random Hexamers (Thermo Scientific Fermentas, Waltham, MA, USA), following supplied protocol. All analyses were performed on Eco Real-Time PCR System (Illumina, San Diego, CA, USA) using Maxima™ SYBR Green/ROX qPCR Master Mix (2×) (Thermo Scientific Fermentas), following supplied protocol. Using cDNA from non-histopathologically changed tissues, standard curves were prepared. All analyzed samples were compared to *ACTB* as a reference gene and non-histopathologically changed tissue as a control, and corrected by reaction efficiency obtained from standard curves. Each measurement was performed in duplicate, in two independent runs. The qPCR results of controls were averaged and used for analysis of all tumor tissues.

## Results

We gathered expression data from eight published microarray studies (Table 1) and performed meta-analysis on a data set derived from 222 tumor and 85 control samples. Seven hundred twenty-five differentially expressed genes were identified for which the summary effect size was lower than −2.5 or greater than 2.5, with FDR less than 0.01 (both cutoffs arbitrarily selected). The top 25 up- and downregulated genes identified in our analysis are listed in Table 2.

**Table 1:** Microarray data sets used in the meta-analysis

Authors	Journal	Year	ID	Array	Criteria	Groups
Cifola et al.	Molecular Cancer	2008	E-TABM-282	133 Plus 2.0	TNM, F grades	ccRCC, normal cortical tissue
Gumz et al.	Clinical Cancer Research	2007	GDS2880	133A	TNM stage 1, 2	ccRCC, normal tissue, the same patient
			GDS2881	133B	TNM stage 1, 2	ccRCC, normal tissue, the same patient
Wang et al.	Nature Medicine	2009	GSE14762	133 Plus 2.0	WHO classification	ccRCC, normal tissue, the same patient
Beroukhim et al.	Cancer Research	2009	GSE14994	133A	Not available	ccRCC, normal tissue, cell lines
Jones et al.	Clinical Cancer Research	2005	GSE15641	133A	TNM, F grades	Clear cell, papillary, chromophobe RCC, OC, TCC, normal tissue
Dalgliesh et al.	Nature	2010	GSE17816	133 Plus 2.0	F grades	ccRCC, normal tissue
			GSE17818	133 Plus 2.0	F grades	ccRCC, normal tissue

**Table 2:** Effect size and FDR values for the 25 top down- and upregulated genes based on differential expression analysis of the tumor and normal tissue

No.	Gene name	Down		Gene name	Up	
		Effect size	FDR		Effect size	FDR
1	TMEM213	11,6732	1,27E−004	HIG2	5,7118	1,71E−016
2	HS6ST2	10,0694	4,55E−006	NDUFA4L2	5,4451	3,74E−015
3	DMRT2	9,5341	7,62E−006	EGLN3	5,3392	1,93E−005
4	UMOD	9,1267	1,30E−008	IKBIP	4,7648	1,61E−033
5	KCNJ1	8,9081	6,19E−006	NNMT	4,7213	2,60E−012
6	CLDN8	8,8482	3,43E−005	VIM	4,7063	1,13E−005
7	KNG1	8,8051	1,65E−006	SPAG4	4,6100	1,12E−004
8	TMEM52B	8,4664	5,69E−006	FUT11	4,5801	2,49E−016
9	ATP6V1G3	8,3852	2,23E−006	PRDX4	4,5110	1,91E−003
10	SERPINA5	7,3164	5,96E−006	PFKP	4,4934	1,62E−004
11	ATP6V0A4	7,0983	5,19E−004	RNF149	4,4640	4,48E−013
12	ATP6V0D2	6,9264	1,00E−006	CDCA2	4,4210	1,00E−016
13	SMIM5	6,9114	1,00E−009	SLC15A4	4,3280	2,09E−045
14	SLC12A1	6,8448	5,70E−004	RNF145	4,2949	7,17E−022
15	HEPACAM2	6,8337	3,88E−007	HK2	4,2734	4,40E−008
16	ATP6V1C2	6,8015	1,40E−005	ENO2	4,2563	1,24E−005
17	FGF9	6,3715	2,21E−005	Hs.201600	4,2467	1,08E−007
18	TFCP2L1	6,3479	1,02E−005	ANGPTL4	4,1907	1,98E−006
19	FAM3B	6,1357	7,68E−007	CXCR4	4,1828	3,04E−006
20	CALB1	6,1284	2,20E−005	Hs.710697	4,1547	6,97E−014
21	FXYD4	6,1276	8,86E−007	MS4A7	4,1397	8,38E−012
22	SLC26A7	5,7771	1,36E−009	TYROBP	4,1360	1,12E−007
23	AQP2	5,7667	1,05E−005	PAG1	4,1228	5,68E−014
24	ERP27	5,6749	2,52E−011	TMSB10	4,1227	6,91E−009
25	TMEM207	5,5021	6,91E−009	SEMA5B	4,1081	9,64E−007

**Fig. 1:**
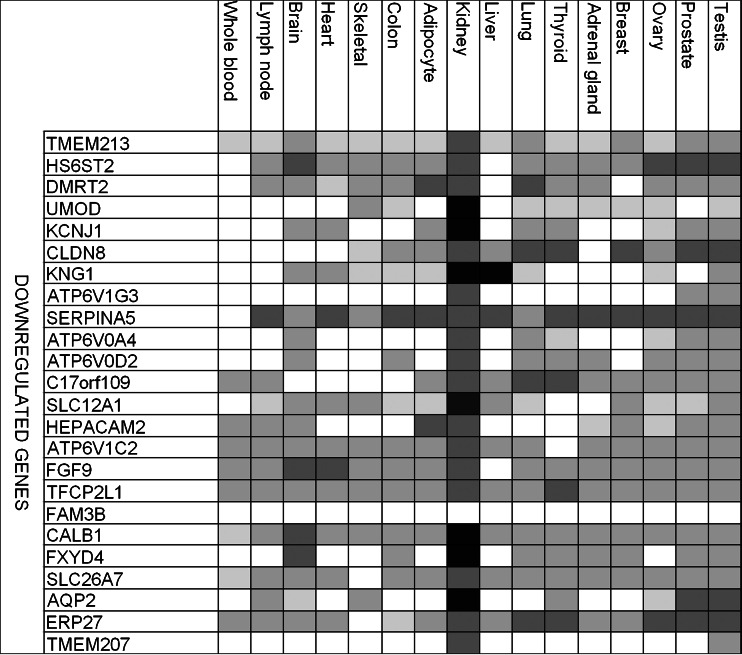
Tissue specific expression of the top 25 downregulated genes identified by the meta-analysis (*darker shade* denotes stronger expression)

**Fig. 2:**
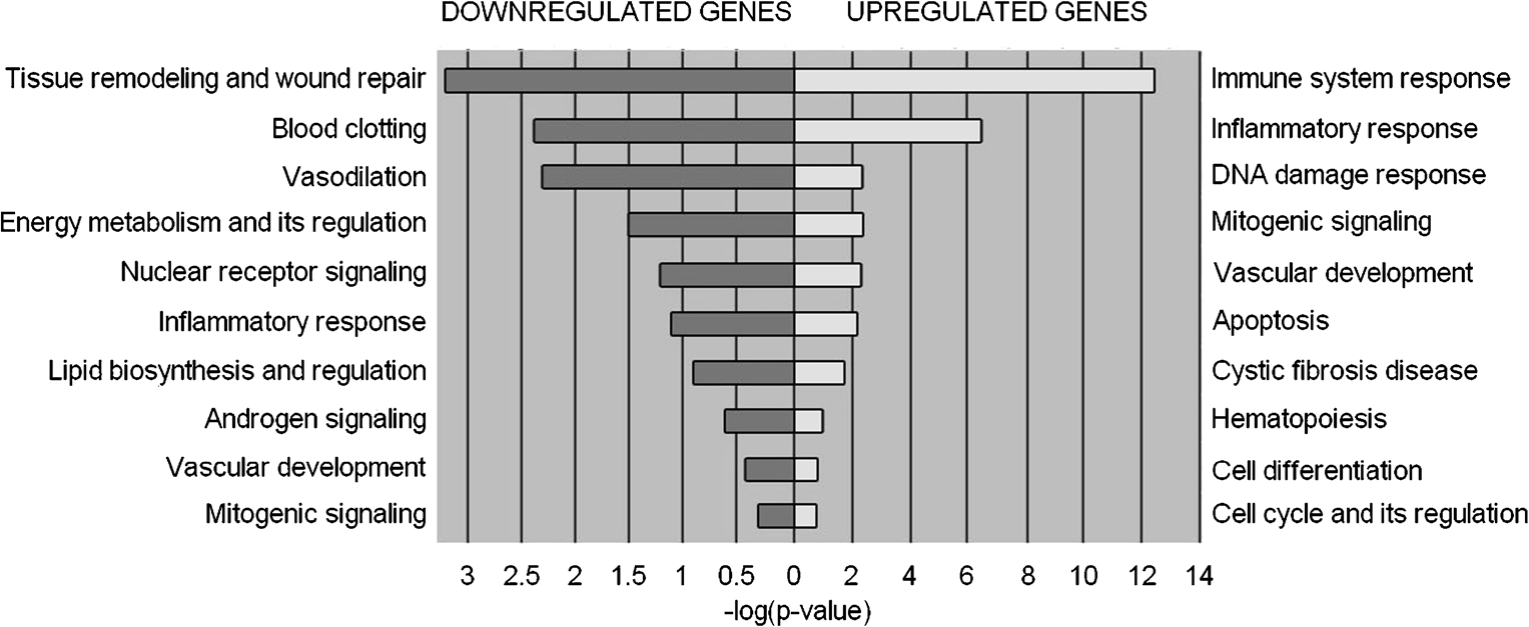
Pathway analysis of down- and upregulated genes (*p* < 0.05)

First, using GeneCards, we investigated expression patterns of the downregulated genes (Fig. 1). Interestingly, 24 of the top 25 downregulated genes were highly expressed mainly in the kidney, with only one gene, *SERPINA5*, being highly expressed additionally in other organs. Limited information was available on the expression pattern of *FAM3B* in any of the listed organs. However, *FAM3B*, also known as PANcreatic DERived factor (*PANDER*), has been recently reported to be decreased in gastric cancers with high invasiveness and metastasis [18]. A few of the downregulated genes were described previously (*UMOD*, *KCNJ1*, or *SERPINA5*), but there is a limited information available on the involvement of, for example, *TMEM213*, *SMIM5*, or *TMEM52B* in ccRCC. In general, we observed that downregulated genes tend to represent biological pathways related to tissue remodeling and wound repair, blood clotting, vasodilatation, and energy metabolism (Fig. 2). Genes involved in tissue remodeling and wound repair (e.g., *CGN*, *TUBAL3*, *EGF*, *PLG*) represent primarily cell adhesion processes such as GAP junction, intercellular channels, extracellular matrix (ECM) remodeling, urokinase-plasminogen activator (PLAU) signaling, and plasmin signaling. The blood clotting pathway is exemplified by genes involved in blood coagulation such as *F11*, *SERPINA5*, *PROC*, and *KNG1*. The genes taking part in vasodilatation (e.g., *XPNPEP2*, *KNG1*, *PLG*) are involved mainly in peptide hormone signal transduction—bradykinin/kallidin pathway. *SREBF2*, *SCAP*, *CASR*, and *NXPH2* represent lipid-associated energy metabolism.

Supporting previously reported data, gene ontology analysis shows a significant clustering of downregulated genes in processes related to ion transport and homeostasis (e.g., cation/anion transport: sodium, potassium, iron), and proper development and function of the kidney nephron, and development of kidney epithelium, renal and urogenital systems (Table 3).

**Table 3:** ccRCC meta-analysis: a list of top 25 down- and upregulated processes according to Metacore classification (M stands for meta-analysis, while T stands for total)

No.	Downregulated				Upregulated			
	Processes	*p* value	No. of genes		Processes	*p* value	No. of genes	
			M	T			M	T
1	Transmembrane transport	1.3E−13	54	1,065	Interferon-gamma-mediated signaling pathway	3.2E−33	30	95
2	Ion transport	3.3E−13	54	1,090	Antigen processing and presentation of exogenous peptide antigen	3.2E−32	33	136
3	Excretion	2.5E−12	15	79	Antigen processing and presentation of exogenous antigen	1.2E−31	33	141
4	Monovalent inorganic cation transport	1.8E−11	28	368	Antigen processing and presentation	3.1E−30	38	231
5	Cation transport	2.1E−11	42	791	Cellular response to interferon-gamma	5.9E−30	31	131
6	Ion transmembrane transport	2.6E−11	35	573	Defense response	2.0E−29	79	1,368
7	Metal ion transport	4.3E−11	37	646	Immune system process	1.7E−28	95	2,040
8	Energy coupled proton transport against electrochemical gradient	4.2E−10	11	50	Positive regulation of immune response	3.4E−28	49	497
9	Transferrin transport	2.9E−09	10	46	Antigen processing and presentation of peptide antigen	5.0E−28	33	179
10	Ferric iron transport	2.9E−09	10	46	Immune response	6.2E−28	71	1,155
11	Anion transport	3.6E−09	20	242	Regulation of immune response	6.8E−28	59	775
12	ATP hydrolysis coupled proton transport	5.7E−09	10	49	Response to interferon-gamma	1.6E−27	31	155
13	Nephron development	2.5E−08	13	108	Innate immune response	2.8E−27	53	625
14	Transport	2.9E−08	106	3,898	Response to stress	2.1E−26	125	3,602
15	Establishment of localization	3.5E−08	107	3,963	Positive regulation of adaptive immune response	2.4E−26	27	113
16	Localization	9.0E−08	121	4,756	Positive regulation of adaptive immune response based on somatic recombination of immune receptors built from immunoglobulin superfamily domains	3.3E−26	26	102
17	Ion homeostasis	1.1E−07	38	902	Positive regulation of immune system process	6.7E−26	56	755
18	Chemical homeostasis	2.2E−07	44	1,164	Cytokine-mediated signaling pathway	7.5E−26	44	434
19	Proton transport	2.3E−07	12	109	Cellular response to cytokine stimulus	9.2E−25	49	592
20	Sodium ion transport	2.6E−07	14	154	Antigen processing and presentation of endogenous peptide antigen	1.3E−24	16	25
21	Hydrogen transport	2.8E−07	12	111	Regulation of immune system process	2.2E−24	68	1,214
22	Organic anion transport	2.9E−07	11	91	Antigen processing and presentation of endogenous antigen	8.1E−24	16	27
23	Cation homeostasis	3.1E−07	29	608	Antigen processing and presentation of exogenous peptide antigen via MHC class I, TAP-independent	8.8E−24	15	22
24	Metanephros development	3.1E−07	12	112	Antigen processing and presentation of exogenous peptide antigen via MHC class I	2.3E−23	25	115
25	Iron ion transport	4.5E−07	10	76	Regulation of adaptive immune response	3.1E−22	28	174

Interestingly, protein products of the downregulated genes were assigned to the three specialized cell compartments: (1) ECM, playing a significant role in the regulation of numerous cellular functions, like cell shape determination, adhesion, migration, proliferation, polarity, differentiation, apoptosis, and wound healing [19, 20]; (2) integral membrane proteins, serving as entry and exit routes for many ions, nutrients, waste products, hormones, drugs, and large molecules (DNA and proteins); (3) membrane vehicles (endosomes and lysosomes) implemented in signal transduction, as well as morphogenetic aspects of normal cell physiology adhesion and migration (Online Resource 1, Table S2).

**Fig. 3:**
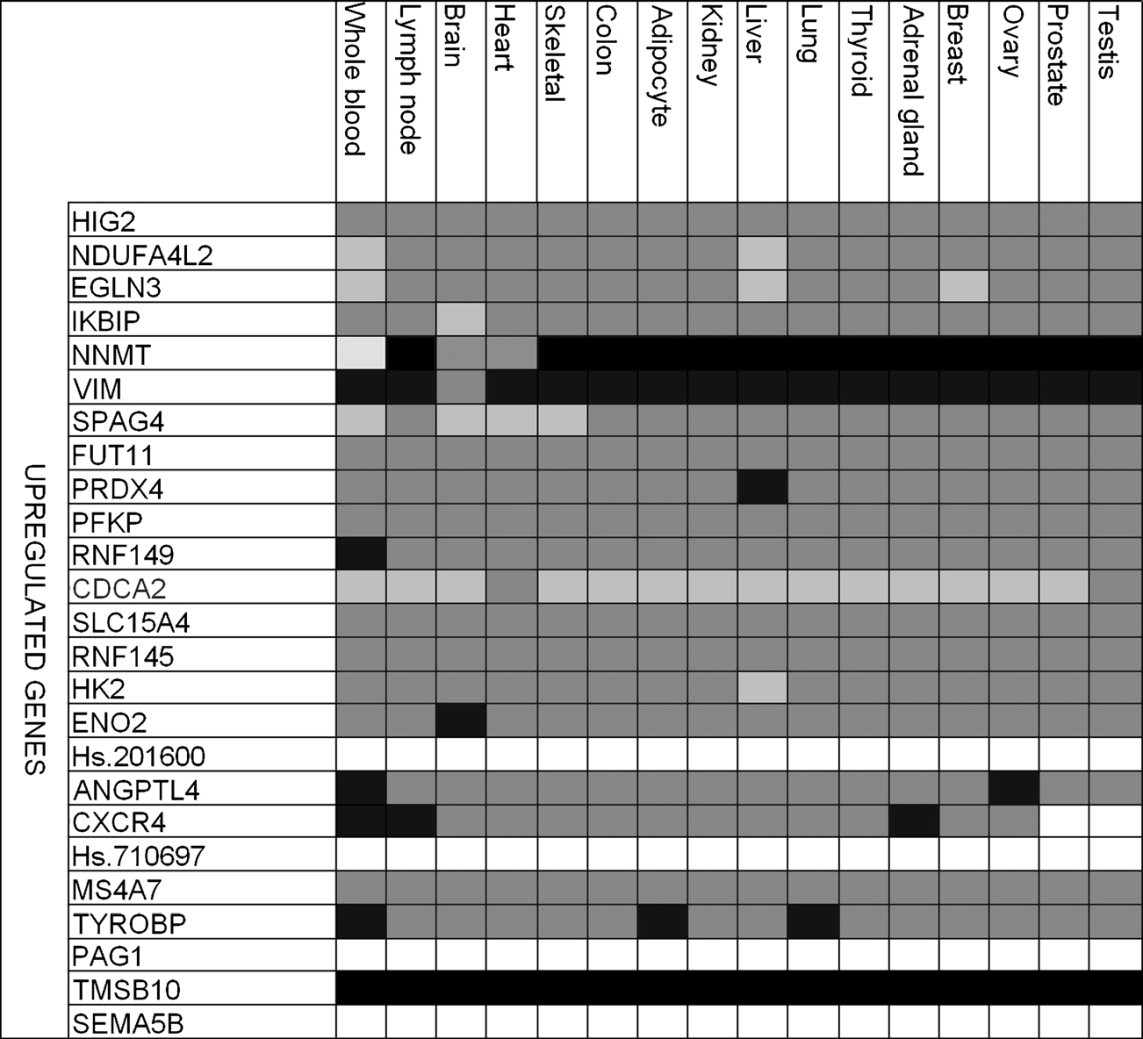
Tissue-specific expression of the top 25 upregulated genes identified by the meta-analysis (*darker shade* denotes stronger expression)

The 25 top upregulated genes are highly expressed in nearly all examined tissues (Fig. 3), except mitotic *CDCA2* and sperm-associated antigen—*SPAG4*. Majority of the most upregulated genes have been previously described (e.g., *HIG2*, *EGLN3*, I*KBIP*, and *VIM*), but we also found a few genes with less well-described function in ccRCC, like alpha-(1,3)-fucosyltransferase 11 (*FUT11*), shown to be expressed in HEK293 cell line, and E3 ubiquitin ligase *RNF149* [21].

All upregulated genes were classified into pathways generally deregulated in cancer: immune system response, inflammatory response, DNA damage response, mitogenic signaling, angiogenesis, and apoptosis (Fig. 2). The immune response is represented by alternative and classical complement pathway (e.g., C3, ITGB2, HLA-DRB), antigen presentation by MHC class I and class II genes, HSP60, HSP70, and TLR signaling pathway. Twenty-eight upregulated genes were assigned to inflammatory response pathways such as HSP60, HSP70, TLR, NF-κB, and TNFR1 signaling pathways, TCR and CD28 co-stimulation in activation of NF-κB (e.g., *UBC*, *LY96*, *TRADD*, *BID*). DNA damage response was represented by a group of inhibitors of apoptosis, which display both anti-apoptotic and pro-survival properties, and their expression can be induced by different cellular stresses such as hypoxia, endoplasmic reticular stress, and DNA damage [22].

The upregulated genes were assigned primarily to the processes of immune response regulation (both positive and negative), cytokine-mediated processes (IFN-γ, cytokine stimulation), and antigen presentation (Table 3). The localization of upregulated gene products was determined mainly as cytoplasmic, but the proteins were also assigned to two additional compartments: cellular membranes and vehicles, supporting the general idea of deregulation of intra- and extracellular signal transduction in ccRCC, similarly to other cancers (Online Resource 1, Table S3).

### Network analysis

**Fig. 4:**
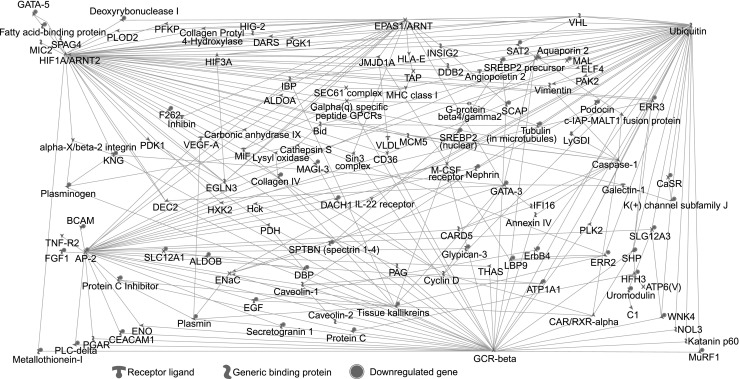
Network analysis of genes deregulated in ccRCC

MetaCore GeneGo program was used to analyze networks of direct interactions between all genes identified in our meta-analysis. Sixty-two differentially regulated genes created a network of interacting genes. The significant interactions between genes (FDR <0.05) are shown in Online Resource 1, Table S4. Our analysis revealed one major network with three distinct central nodes: UBC, AP-2, and *GCR-β* located centrally and with extensive connections to other genes (Fig. 4). Notably, there were several nodes (plasmin, caspase 1, ENaC, protein C inhibitor, and tissue kallikreins) interconnecting central networks. Inclusion of *VHL*, *HIF1A*, *EPAS1*, and *HIF3A* in the analysis resulted in addition of the next 53 deregulated genes to the network (e.g., *PFKP*, *HIG2*, *DARS*, *HLA-E*, *JMJD1A*). We observed that HIF1A joined the subset of central nodes to create a comprehensive network with others genes (e.g., *PFKP*, *HIG-2*, and *KNG*). EPAS1 was shown to interact with 23 genes (e.g., *EGLN3*, *HLA-E*, and *VEGFA*) (Fig. 4).

### Validation of the expression of the most deregulated genes in independent sample set

We set to validate the expression of the seven most down- and the seven most upregulated genes in 32 tumor samples derived from 31 ccRCC patients of Greater Poland. We concentrated on candidate genes not previously reported due to differences in methodology used or not discussed by others: *HIG2*, *NDUFA4L2*, *EGLN3*, *FUT11*, *PRDX4*, *PFKP*, *RNF149*, *TMEM213*, *HS6ST2*, *DMRT2*, *CLDN8*, *TMEM52B*, *ATP6V1G3*, and *ATP6V0A4*. First, tumor samples were examined for *VHL*, *HIF1A*, and *EPAS1* expression. Similarly to previously reported data, we observed 20–50 % reduction of *VHL* mRNA levels as compared to healthy tissue (data not shown [23]).

**Fig. 5:**
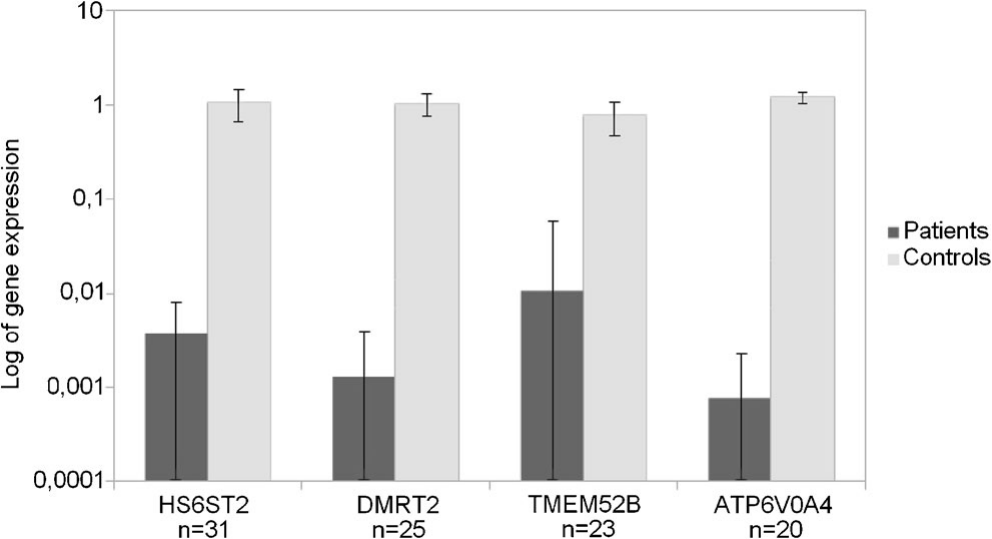
Downregulation of validated genes in the cohort of 31 ccRCC patients

All seven genes found as most downregulated in our meta-analysis had decreased expression in all tested tumors. Heparan sulfate 6-O-sulfotransferase 2 (*HS6ST2*) was downregulated 267-fold on average and detected in 31 (96.88 %) samples (Fig. 5). Average 772-fold downregulation of Doublesex and Mab-3 Related Transcription Factor 2 (*DMRT2*) was found in 25 (78.12 %) tumors. We detected on average 94-fold lower expression of *TMEM52B* in 23 (71.88 %) samples. Transmembrane protein 213 (*TMEM213*) and Claudin 8 (*CLDN*8) were detected only in three (0.94 %) and four (1.25 %) tumor specimens, with average folds of 1,066 and 226, respectively. The expression of *ATP6V0A4* was found in 20 (62.5 %) tumors and was down 1,308 times on average, while the expression of *ATP6V1G3* was undetectable in all samples.

**Fig. 6:**
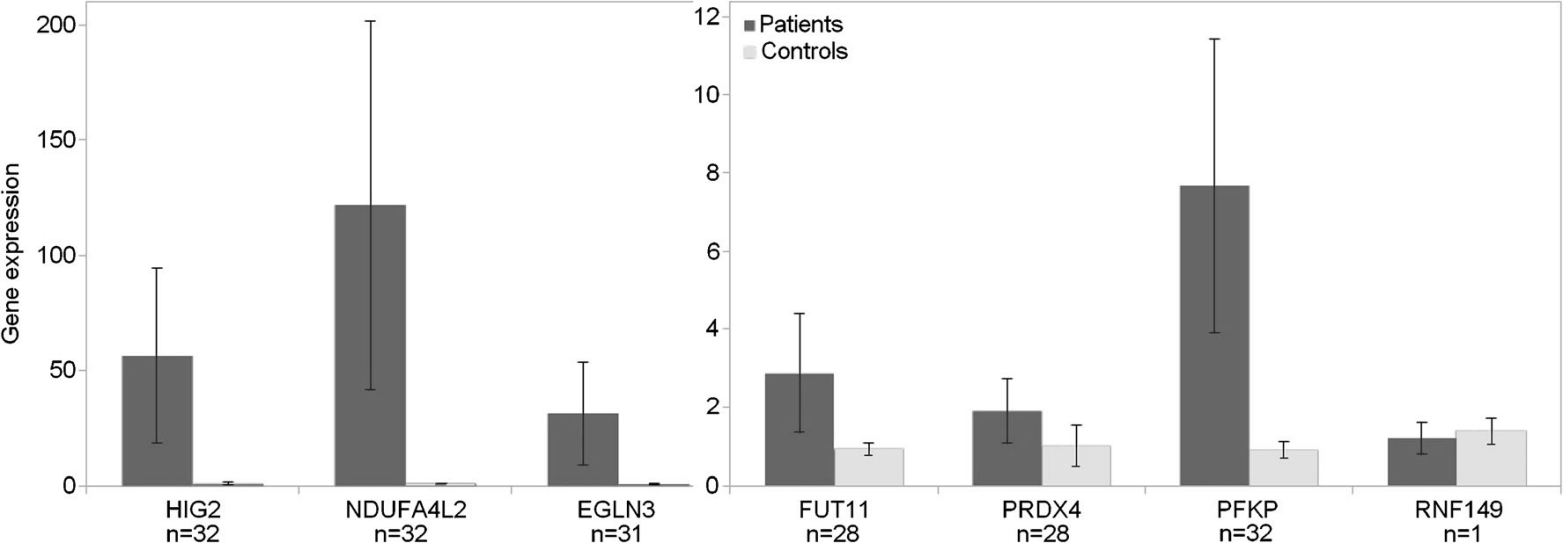
Upregulation of validated genes in the cohort of **32** ccRCC patients

The relative expression of hypoxia inducible lipid 2 (*HIG2*), found upregulated in our meta-analysis, was increased, on average 56-fold, in all tumors tested as compared to the healthy tissue (Fig. 6). The upregulation (on average 122-fold) of mitochondrial NADH dehydrogenase [ubiquinone] 1 alpha subcomplex, 4-like 2 (*NDUFA4L2*) was found in all tumors, whereas EGL9 homolog 3 (*EGLN3*) was overexpressed in 31 (96.88 %) samples and 32-fold on average. The mean upregulation of phosphofructokinase (*PFKP*) and fucosyltransferase 11 (*FUT11*) was equal to 7.6 and 2.9, respectively (overexpressed in 32 (100 %) and 28 (87.5 %) samples). The expression of Peroxiredoxin 4 (*PRDX4*) was up in 28 (87.5 %) tumors and increased 1.92-fold on average, while *RNF149* was overexpressed approximately 2.26-fold in one tumor specimen. The increased expression of majority of validated upregulated genes (*n* = 5, except *HIG2* and *RNF149*), correlated with the expression of *VHL*, *HIF1A*, and EPAS1, with highest correlation coefficient for *FUT11* (0.71), *EGLN3* (0.60), *PFKP* (0.58), and *NDUFA4L2* (0.48), and lowest for *PRDX4* (0.39), indirectly suggesting their dependence on *VHL*, *HIF1A*, and *EPAS1*. Further, we investigated if the expression of validated genes could be used as a predictor of disease progression using the same group of 31 ccRCC patients. Patient characteristics are shown in Table 4. Due to incomplete clinical data, four patients were excluded from the analysis. First, using forward logistic regression, we found that, out of all genes tested, *FUT11*'s expression was associated with disease progression (*p* = 0.025, OR = 0.392, 95 % CI = (0.173–0.891)) (*ATP6V1G3*, *TMEM213*, and *CLDN8* were excluded due to strong downregulation). Combined analysis of clinical and molecular parameters showed that *FUT11* remained a significant parameter in the model (*p* = 0.042, OR = 0.215, 95 % CI = (0.049–0.949)), together with TNM (*p* = 0.024, OR = 2.379, 95 % CI = (1.124–5.036)) and diabetes (*p* = 0.047, OR = 0.003, 95 % CI = (0.000–0.924)).

**Table 4:** Characteristics of patient validation cohort

Variable	Patients
No. of cases	31
Age at surgery:	
Mean	65
Range	31–80
Sex:	
Male	18
Female	13
Diabetes	5
Average tumor size (mm):	
Mean	57
Range	25–128
Grade:	
G1	1
G2	15
G3	8
G4	7
Stage:	
I	12
II	2
III	8
IV	10
T:	
2	8
3	12
4	5
6	3
7	2
9	1
N:	
0	28
1	3
M:	
0	22
1	9

**Fig. 7:**
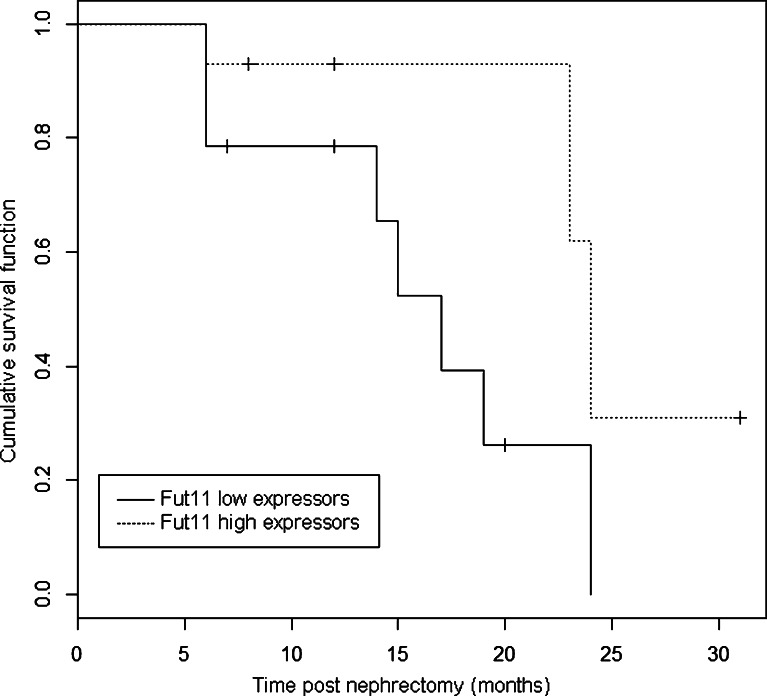
Kaplan–Meier plot for low and high FUT11 expressors

Secondly, we examined the correlation between *FUT11* mRNA levels and disease course up to 31 months post-nephrectomy. The patients were divided into two groups: individuals with high *FUT11* expression (values greater than the average expression in tumors, *n* = 14) and low *FUT11* expression (*n* = 14). We observed that majority (12 out of 17) of patients with non-symptomatic disease course displayed high *FUT11* expression. We found inverse correlation between the two variables (linear correlation coefficient *ρ* = −0.51), what, taking under consideration the fact that ccRCC is a complex, multigenic disease, may suggest the importance of *FUT11* expression in the ccRCC pathology. In Kaplan–Meier survival analysis, the association between *FUT11* expression and non-sympomatic disease course did not reach statistically significant value, most likely to due to relatively small sample set analyzed (see Fig. 7). The results of univariate and multivariate survival analyses with Cox regression model to assess *FUT11*'s input independently of other predictor variables are shown in Table 5.

**Table 5:** Univariate and multivariate analyses of the effect of *FUT11* expression, and of the sex, age, tumor size, (F)uhrman grade, symptoms, and pT parameters, on disease progression

Variable	Univariate analysis	Multivariate analysis		
	*p* value	*p* value	HR	95 % CI
Sex	0.311	0.076	2.09	0.8–81.95
Age	0.613	0.634	−0.41	0.12–3.61
Tumor size	0.436	0.067	−5.25	0.00–1.45
F grade	0.72	0.478	1.39	0.08–182.95
Fut11	0.075	0.464	−1.29	0.00–8.62
Symptoms	0.001	0.078	4.17	0.62–6684
pT	0.039	0.039	3.11	1.17–434.64

## Discussion

In the present study, we performed a meta-analysis of ccRCC gene expression data derived from public repositories and executed extensive analyses of pathways, processes, and cellular localization of the differentially expressed genes. In our meta-analysis, we implemented the methodology proposed by Ramasamy et al. [12], based on estimation of effect size, in contrast to standard fold change analysis. Effect size measures the overlap of the distributions of signal intensity in cases and controls for each gene, but does not impart how much a gene's expression has changed between cases and controls in terms of fold change. And since effect size does not depend on actual expression values, but on a relationship between them, it is suitable for the analysis of data derived from different experiments, carried out in different conditions and on different platforms.

Using this approach, we found 725 genes deregulated in the tumor tissue. The generated list included genes extensively described in previous reports such as downregulated (*UMOD*, *KNG1*, *SERPINA5*, *KCNJ1*) and upregulated (*EGLN3*, *VIM*, *HIG2*) [24–27], but it also contained genes of less recognized function in ccRCC pathogenesis, represented by *TMEM21*3 and ATPases: *ATP6V1G3*, *ATP6V0A4*, *ATP6V0D2*.

In line with previous reports, the downregulated genes were mapped to biological processes and pathways essential for proper kidney function (e.g., ion transport and homeostasis) and development (e.g., nephron and kidney epithelium development) [26], whereas the upregulated genes were classified into pathways known to be deregulated in cancer: immune system response, inflammatory response, DNA damage response, angiogenesis, and apoptosis [28].

Network analysis highlighted a few important genes as points of functional convergence, including those recently described in ccRCC: *EGLN3* [29], *AP-2*, *NR3C1*, kallikreins, and well recognized *HIF1A*, *EPAS1*, and genes encoding ubiquitin. Interestingly, only 115 of the 725 deregulated genes were included in the networks, not only supporting the importance of VHL/HIF pathways in ccRCC pathology but also highlighting the significance of additional processes in the development of this disease, as suggested by others [23, 30].

Regression analysis of expression of the validated genes in combination with clinical data showed potential applicability of *FUT11* expression as a marker of non-symptomatic disease. Interestingly, we observed correlation of high *FUT11* expression with non-symptomatic disease course.

*FUT11* belongs to a family of fucosyltransferases—globular type II transmembrane Golgi-resident proteins. Their function is to catalyze the transfer of α-l-fucose from GDP-Fuc onto N- and O-linked glycans, free oligosaccharides, lipids, or directly onto proteins; however, the fucosyltransferase activity has not been confirmed for *FUT11* [31]. Fucose, as a constituent of oligosaccharides, is associated with cancer and inflammation [32].

Currently, there is no information available concerning the role of *FUT11* in ccRCC, but its upregulation has been detected in additional microarray data sets [33, 34]. *FUT11* has also been found upregulated in autosomal dominant polycystic kidney disease expression data [35]. First functional data were provided by Groux-Degroote et al. [33], who showed that IL-6 and IL-8 have stimulatory effect on expression of *FUT11*, and *FUT11* may be involved in the biosynthesis of sialyl-Lewis^x^ and 6-sulfo-sialyl-Lewis^x^ epitopes in the bronchial mucins in inflammatory mucosae of cystic fibrosis patients. Lewis epitopes are crucial for leukocyte homing and extravasation process, thus are essential for lymphocyte maturation and the function of immune system [36]. On the other hand, IL-6 plays an important role in the immune defense mechanism and cell growth and differentiation modulation in numerous malignancies [37]. It has been observed that expression of fucosylated oligosaccharides changes in cancer and inflammation (e.g. [31]), also in ccRCC [38]; hence, detection of *FUT11* upregulation in our meta-analysis and in ccRCC tumors may link *FUT11* to ccRCC development and progression.

Although our preliminary data suggests involvement of *FUT11* in ccRCC progression, our findings require independent validation on additional large sample sets. Further functional studies are needed to acquire more detailed knowledge on the role of this fucosyltransferase in ccRCC development.

## Electronic supplementary material

Below is the link to the electronic supplementary material.

ESM 1 (XLS 49 kb)
